# Modelling inner ear development and disease using pluripotent stem cells – a pathway to new therapeutic strategies

**DOI:** 10.1242/dmm.049593

**Published:** 2022-11-04

**Authors:** Keeva Connolly, Anai Gonzalez-Cordero

**Affiliations:** ^1^ Stem Cell Medicine Group, Children's Medical Research Institute, Westmead, 2145 NSW, Australia; ^2^ School of Medical Sciences, Faculty of Medicine and Health, University of Sydney, Westmead, 2145 NSW, Australia

**Keywords:** Inner ear, Pluripotent stem cells, Disease modelling, Development

## Abstract

The sensory epithelia of the mammalian inner ear enable sound and movement to be perceived. Damage to these epithelia can cause irreversible sensorineural hearing loss and vestibular dysfunction because they lack regenerative capacity. The human inner ear cannot be biopsied without causing permanent damage, significantly limiting the tissue samples available for research. Investigating disease pathology and therapeutic developments have therefore traditionally relied on animal models, which often cannot completely recapitulate the human otic systems. These challenges are now being partly addressed using induced pluripotent stem cell-derived cultures, which generate the sensory epithelial-like tissues of the inner ear. Here, we review how pluripotent stem cells have been used to produce two-dimensional and three-dimensional otic cultures, the strengths and limitations of these new approaches, and how they have been employed to investigate genetic and acquired forms of audiovestibular dysfunction. This Review provides an overview of the progress in pluripotent stem cell-derived otic cultures thus far, focusing on their applications in disease modelling and therapeutic trials. We survey their current limitations and future directions, highlighting their prospective utility for high-throughput drug screening and developing personalised medicine approaches.

## Teaser

**Summary:** Pluripotent stem cell-derived differentiation cultures can recapitulate inner ear developmental progression *in vitro*, and their utility for applications in disease modelling and therapeutic trials will likely improve as ongoing research overcomes technical challenges.

## Introduction

**Fig. 1.**
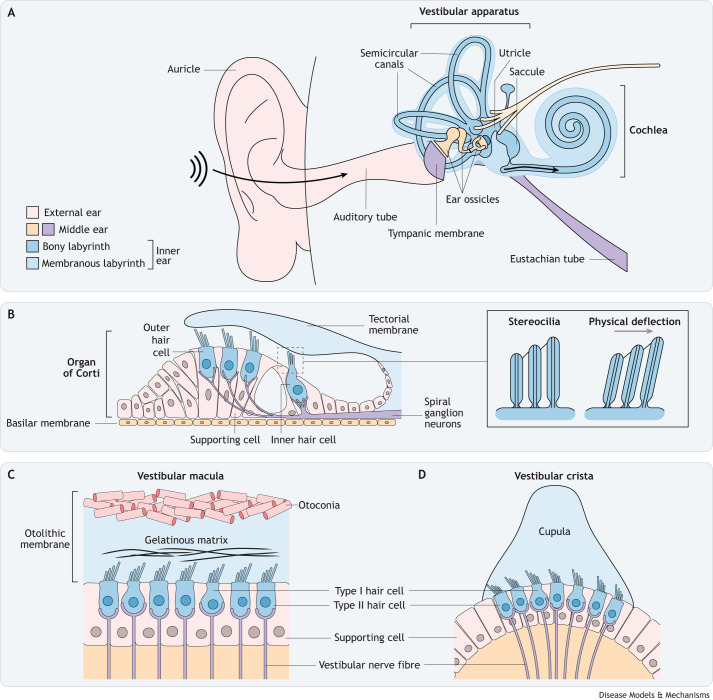
**An overview of the human ear and inner ear.** (A) The mammalian ear, showing the external, middle and inner ear. The external ear is made up of the auricle, auditory tube and tympanic membrane. The middle ear includes the ear ossicles and eustachian tube. The inner ear is composed of the cochlea, vestibular apparatus (including the utricle, saccule and semi-circular canals) and endolymphatic sac. Arrows show stimuli entering the external ear, stimulating the ossicles of the middle ear and reaching the inner ear. (B) A cross section of the sensory organ of the cochlea, the organ of Corti, showing three parallel rows of innervated outer hair cells, one parallel row of innervated inner hair cells, and surrounding supporting cells, underneath an overlying tectorial membrane. Highlighted panel shows a magnification of the cochlear hair cells’ stereocilium. Soundwaves and movement physically deflect the stereocilia towards the tallest stereocilium. (C) A cross section of the vestibular macula, including innervated type I and II hair cells and neighbouring supporting cells embedded in the gelatinous matrix of the otolithic membrane, which is overlaid with crystalline otoconia. (D) A cross section of the vestibular crista, including innervated type I and II hair cells and neighbouring supporting cells embedded in the gelatinous cupula.

Hearing and balance are underpinned by the translation, in the inner ear, of physical stimuli into electrical impulses, which are processed in the brain. This phenomenon, termed mechanotransduction, occurs in the snail-shaped cochlea, which detects sound, and in the labyrinthine vestibular apparatus, which detects movement and gravity (Fig. 1) (reviewed in Caprara and Peng, 2022; Ó Maoiléidigh and Ricci, 2019). In both organs, mechanotransduction takes place in hair cells, so named for the thin, hair-like protrusions of actin-based stereocilia at their apical surface. These specialised stereocilia are physically deflected in response to soundwaves or movement, opening mechanically gated ion channels located at their tips and triggering generation of a mechanoelectrical transducer (MET) current (Beurg et al., 2009; Corey and Hudspeth, 1983).

Human hair cells cannot restoratively regenerate once damaged, leading to irreversible impairments in hearing and balance. Factors that contribute to hair cell loss include ageing, exposure to noise and ototoxic drugs (see Glossary, Box 1), genetic mutations and infectious diseases. Hearing loss alone is estimated to affect 20% of the global population, including 430 million people with disabling hearing impairment (World Health Organization, 2021). Research into the mechanisms that underpin hair cell development, dysfunction and death *in situ* has historically faced a logistical bottleneck. This is because the human inner ear is somewhat inaccessible, located within a bony encasement, and because auditory hair cells cannot regenerate, rendering biopsies unfeasible without causing lasting damage. The use of animal models, particularly the mouse, in which cochlear and vestibular anatomy is largely conserved and which bears similar developmental transcriptional profiles (Roccio et al., 2018), has significantly advanced research in this field. However, animal models bear some key differences to their human counterparts. Knockouts of deafness-associated gene orthologues in mice do not always correlate to expected pathological phenotypes, as has been observed for *Crym* (Suzuki et al., 2007) and *Gjb3* (Plum et al., 2001), indicating differences in gene function, expression and/or redundancy. Similarly, postnatal hair cell regenerative capacity varies between species; in zebrafish, it is lifelong (Ma et al., 2008; Pinto-Teixeira et al., 2015), and, in mice, it is briefly retained in neonatal cochlea (Cox et al., 2014). Inner ear accessibility in mammalian model organisms is also hampered by the same anatomical constraints encountered in humans. Although mouse explant cultures can be used to maintain cochlear and vestibular cells *in vitro*, inner ear tissues can be difficult to dissect, and cultures are limited in longevity, with hair cell numbers deteriorating rapidly after 12 days (Nayagam et al., 2013).

**Box 1. Glossary**

**Cochlin (*COCH*):** gene encoding an extracellular matrix protein linked to autosomal-dominant deafness.

**Cochleostomy:** a procedure in which an opening is drilled into the cochlea to allow direct access to the sensory epithelia.

**Direct transdifferentiation:** a process in which a cell adapts from one cell type to another without re-entering the cell cycle.

**Deafness, autosomal dominant 11 (DFNA11):** autosomal-dominant non-syndromic hearing impairment caused by mutations in *MYO7A*, encoding a myosin motor protein.

**Deafness, autosomal recessive 2 (DFNB2):** autosomal-recessive non-syndromic hearing impairment caused by mutations in *MYO7A*, encoding a myosin motor protein.

**Deafness, autosomal recessive 9 (DFNB9):** autosomal-recessive non-syndromic hearing impairment caused by mutations in *OTOF*, encoding a calcium ion sensor involved in regulation of neurotransmitter release.

**Ectoderm germ layer:** one of the three primordial germ layers produced during gastrulation. It gives rise to surface tissues, such as the skin, and neural tissues, including the central nervous system.

**Espin (*ESPN*):** gene encoding an actin-bundling protein linked to autosomal-recessive deafness.

**FM1-43X dye:** a fluorescent styryl dye used to visualise membrane dynamics. In hair cells, dye uptake reflects endocytosis to replenish the synaptic membrane, indicating synaptic release induced by mechanotransduction.

**Greater epithelial ridge:** a developmentally transient population of cochlear cells immediately bordering the inner hair cells and neighbouring supporting cells in the neonatal organ of Corti.

**Inner cell mass:** a cellular mass with pluripotent capacity inside the blastocyst (pre-implantation) staged embryo, which will give rise to epiblast and hypoblast, with the former giving rise to the three primordial germ layers.

**Mitotic regeneration:** a process in which a cell re-enters the cell cycle to produce progeny that may differentiate into a different cell type.

**Myoclonic epilepsy with ragged-red fibres (MERRF) syndrome:** a mitochondrial disorder with diverse symptoms of variable severity, including myoclonic twitches or jerks, seizures, vision loss, deafness and dementia.

**Otoconia:** calcium carbonate-based crystal structures that blanket and give weight to the otolithic membrane in the vestibular maculae.

**Ototoxic drugs:** therapeutic substances, including certain antibiotics (e.g. aminoglycosides) and chemotherapeutics (e.g. cisplatin), which accumulate and induce damage in the inner ear.

**Pendred syndrome:** an inherited condition characterised by congenital bilateral sensorineural hearing loss, thyroid goitre and, in some cases, vestibular dysfunction.

**Ribbon synapse:** a type of synapse in which synaptic vesicles are tethered to a ribbon-like structure within the pre-synaptic cell, enabling rapid release of neurotransmitters in response to changes in membrane potential.

**Round window:** a membrane-covered opening between the middle and inner ear facilitating soundwave conduction by enabling movement of the inner ear fluids.

**Usher syndrome:** an autosomal-recessive disease characterised by deafness, blindness and variable vestibular dysfunction.

The differentiation potential of pluripotent stem cells (PSCs), including embryonic stem cells (ESCs) and induced pluripotent stem cells (iPSCs), into various tissues of the body provides a way to circumvent some of these challenges and bridge the gap between animal and human systems. The temporally controlled addition of exogenous signalling factors and/or small molecules to undifferentiated PSCs induces an artificial developmental niche that can induce stepwise otic differentiation *in vitro*. Such cultures produce inner ear sensory cell types with demonstrable functionality that can be maintained for periods of weeks to months (Mattei et al., 2019; Ohnishi et al., 2015; Oshima et al., 2010; Tang et al., 2019). This renewable source of auditory and vestibular tissue and cell types makes these culture platforms valuable for studying otic development, function and disease in living tissue.

In recent decades, PSC-derived cultures have been rendered three dimensional (3D) through the creation of organoids, in which cells differentiate and self-organise in suspension to resemble miniaturised organs. Inner ear organoid culture protocols can generate sensory epithelia-like structures that contain innervated hair cells with mature electrophysiological profiles in conjunction with other surrounding tissue types (Koehler et al., 2013, 2017; Liu et al., 2016). Their morphological and functional resemblance to the *in vivo* inner ear makes these organoids a promising tool for disease modelling and for testing new therapeutics.

Here, we review progress in the field of PSC-derived inner ear cultures of the sensory epithelia, following a brief introduction to mammalian inner ear development. We compare the different approaches used to generate these remarkable models, and discuss how they have been used so far to further our understanding of auditory diseases and to trial therapeutic strategies. Finally, we survey the current challenges facing these cultures and explore how overcoming these will expand the possibilities for disease modelling and therapeutic trials in the inner ear.

## The inner ear and its functional attributes

The mammalian ear contains three parts: the outer ear, the middle ear and the inner ear (Fig. 1A). The outer ear is the external part composed of the auricle, the auditory tube and the tympanic membrane, also known as the eardrum. The middle ear is formed by the ossicles and the eustachian tube. The inner ear consists of a continuous, fluid-filled membranous labyrinth that houses the sensory tissues of the cochlea and vestibular apparatus, which are embedded within an intricate bony labyrinth (Fig. 1A).

The cochlear sensory epithelium is located in the organ of Corti and contains an array of innervated hair cells and supporting cells (Fig. 1B). Sound waves are funnelled through the outer ear canal to the tympanic membrane and are amplified by the three ossicle bones of the middle ear, and then pass into the inner ear. Here, they travel through lymphatic fluid to the basilar membrane, on which the organ of Corti sits, to a point that correlates with sound wave frequency (Stakhovskaya et al., 2007). Vibrations at the basilar membrane reverberate upwards through the organ of Corti to the hair cell stereocilia (Fig. 1B).

The vestibular apparatus consists of two bulbous chambers, the utricle and saccule, which house the maculae (Fig. 1C), and three semi-circular canals, which contain the cristae (Fig. 1D). When the head moves along horizontal or vertical planes, the sensory epithelium in the maculae move in tandem, but the overlying otolithic membrane, which is weighted down by the otoconia (Box 1), remains in place. This movement exerts a shearing force on the protruding stereocilia (Dohlman, 1971; Jaeger et al., 2002). In the semi-circular canals, rotational movements are registered by the cristae because the surrounding endolymph remains static, exerting angular forces on the cupula, which are conveyed to the stereocilia (McLaren and Hillman, 1979).

These stereocilia are organised in a staircase formation, with parallel rows of increasing height interconnected by tip links (Fig. 1B). In response to vibrations, the shorter stereocilia are deflected towards the taller stereocilia, and displacement of the tip links creates a ‘pulling’ force that induces the opening of mechanically gated MET channels (Beurg et al., 2009; Corey and Hudspeth, 1983). Channel opening allows an influx of cations, such as Ca^2+^ and K^+^, from the surrounding endolymph to enter at the site where the tip link meets the shorter stereocilium (Beurg et al., 2009), generating a MET current that is transmitted through the spiral ganglion neurons towards the auditory cortex.

In vestibular hair cells, rows of stereocilia increase stepwise in height towards a taller tubulin-based kinocilium. Head movements result in stereocilia deflection, tip link tensioning and MET channel opening, to produce a MET current that is conducted through the vestibular nerve.

## The complex development of the inner ear

**Fig. 2.**
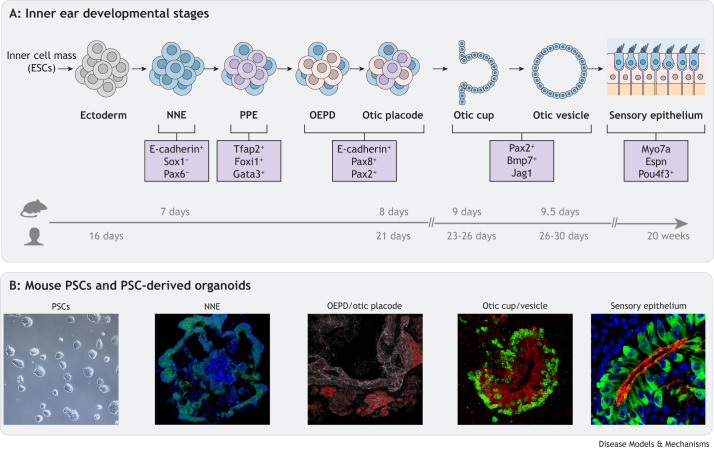
**Otic developmental trajectory and pluripotent stem cell differentiation time course.** (A) Schematic representation of the various stages of inner ear development shown in parallel with a timeline of mouse and human otic development *in vivo*. Important markers that delineate each state are noted. Time points are given as days post conception. (B) Representative confocal images of mouse PSCs and their derivative inner ear organoids, showing the different stages of development depicted in A. The NNE is E-cadherin^+^ (green) and Sox1^−^ (red); nuclei are shown in blue. The OEPD/otic placode shows E-cadherin in grey and Pax8 in red. The otic cup/vesicle shows Sox2 in green and Jag1 in red. Finally, the sensory epithelium shows Myo7a in green, phalloidin in red and nuclei in blue. ESC, embryonic stem cell; NNE, non-neural ectoderm; OEPD, otic-epibranchial progenitor domain; PE, preplacodal ectoderm; PSC, pluripotent stem cell.

Numerous signalling interactions during mammalian embryogenesis shape and pattern the inner ear. The embryonic ectoderm germ layer (Box 1), derived from the inner cell mass (Box 1) at the gastrula embryonic stage produces the non-neural ectoderm (NNE), which is distinguishable from the bordering hindbrain neuroectoderm by expression of the intercellular adhesion molecule E-cadherin [cadherin 1 (Cdh1)] (Fig. 2A) (Nandadasa et al., 2009) and absence of Pax6 and Sox1, a transcription factor driving neuroectoderm specification (Zhang et al., 2010). Subsequent expression of transcription factors Foxi1, Gata3, Tfap2a and Tfap2c drive specification of the pre-placodal ectoderm (PPE) (Kwon et al., 2010), which gives rise to the cranial sensory structures. From the PPE, otic-specifying transcription factors Pax2 and Pax8 induce the generation of the otic-epibranchial progenitor domain (OEPD) and subsequent otic placode (McCarroll et al., 2012). The otic placode invaginates to create the otic cup, which eventually becomes pinched off to produce the otic vesicle. Cells within these structures generally express markers Pax2, Bmp7 and Jag1 (Li et al., 2003). Morphogenic signalling, including Wnt, sonic hedgehog (SHH), retinoic acid (RA), bone morphogenetic protein (BMP) and Notch pathways, specifies the otic vesicle's axes, the vestibular and cochlear structures, and generates the sensory epithelia (Bok et al., 2011; Ohyama et al., 2010; Petrovic et al., 2014; Riccomagno et al., 2005).

As the mechanics of these developmental processes have come to light, it has become possible to mimic *in vivo* molecular and genetic cues to induce differentiation *in vitro* (Fig. 2B). Combining these findings with PSCs has enabled recapitulation of the otic developmental pathway to produce auditory and vestibular sensory cell types *in vitro*, providing new opportunities to study the human inner ear without the need for tissue dissection or extraction, as we discuss in the following sections.

## Evolution of PSC-derived *in vitro* models

The capacity of PSCs to differentiate along multiple cell fate lineages underlies their utility as a versatile research tool. In the case of inner ear sensory cells, PSCs have been induced using protocols that manipulate the signalling pathways active during otic development, such as FGF, Wnt and BMP, to drive differentiation along the otic lineage (Li et al., 2003; Oshima et al., 2010; Ronaghi et al., 2014). As the field has progressed, earlier protocols that made use of mouse-derived PSCs have been adapted for human cells (Chen et al., 2012; Ronaghi et al., 2014), and foundational work in two-dimensional (2D) monolayer-based cultures has been translated into 3D, enabling the formation of complex organoids that bear greater structural similarity to the developing inner ear *in vivo* (Koehler et al., 2013, 2017) (Fig. 2A,B).

## The original 2D culture systems

**Fig. 3.**
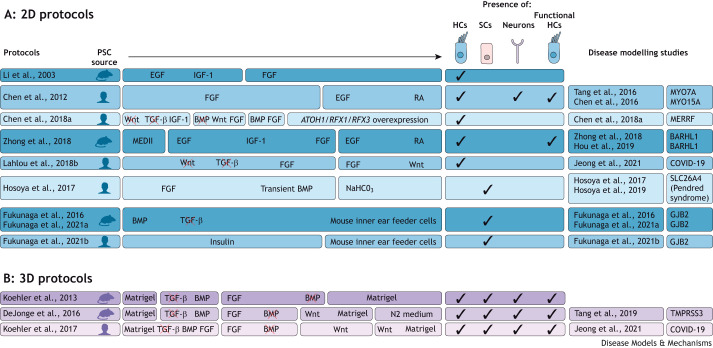
**Summary of differentiation protocols used for disease modelling.** (A,B) Overview of selected studies using 2D (A) and 3D (B) PSC-derived inner ear culture protocols, including the source of PSCs, and the targeted activation and inhibition of signalling systems. Ticks demonstrate the cell type generated and the derived cell types shown to be functional. Different protocols were used in disease modelling studies of specific inner ear diseases. HCs, hair cells; MEDII, conditioned medium from the hepatocellular carcinoma cell line HepG2; PSC, pluripotent stem cell; SCs, supporting cells.

In the first inner ear differentiation protocol, mouse ESCs were aggregated in suspension to form embryoid bodies (EBs), then plated in adhesive monolayer culture (Fig. 3A) and induced to develop into PPE progenitor cells by exposure to epidermal growth factor (Egf) and insulin-like growth factor 1 (Igf-1) (Li et al., 2003). The subsequent addition of fibroblast growth factor 2 (Fgf2) elicited an expression profile that is typical of cells present in the otic placode and vesicle (Pax2^+^/Bmp7^+^/Jag1^+^). A period of self-directed development resulted in the cursory expression of hair cell marker proteins, including myosin VIIa (Myo7a), Espn and Pou4f3 (Brn3.1).

## Exogenous signalling molecules and co-culture methods driving otic development

The work of Li and colleagues laid the foundation for further research into alternative signalling pathway manipulations and culture methods to refine the control of each otic developmental transition. For example, Oshima et al. (2010) combined the inhibition of Wnt and transforming growth factor β (Tgf-β) pathways with the addition of Igf-1 to suppress mesoderm differentiation and promote ectoderm specification (Conlon et al., 1994; Liu et al., 1999). The resulting progenitor cells were treated with Fgfs and co-cultured with chicken utricle stromal cells to induce differentiation of hair cell-like cells bearing organised cadherin-23^+^ stereocilia and exhibiting electrophysiological profiles similar to those of immature hair cells (Oshima et al., 2010). Subsequent work found that this co-culture system could by bypassed by using conditioned media obtained from stromal cell culture, which induced hair cell-like cells with rudimentary stereocilia structures able to take up FM1-43FX dye (Box 1), indicating MET channel functionality (Ouji et al., 2012).

Protocols for deriving human inner ear cells *in vitro* were founded on these original mouse PSC protocols. Ronaghi et al. (2014) utilised a similar IGF-1 addition/Wnt inhibition/TGF-β inhibition approach as Oshima et al. (2010) to drive ectodermal differentiation in human ESCs. The resulting cells were supplemented with FGFs and combined with Wnt signalling reactivation and BMP signalling modulation to promote the development of the NNE into the OEPD and otic placode. In human iPSC-derived cultures, this progression was observed when treated with the same signalling factors (with the exception of IGF-1) and with RA, which was necessary to elicit expression of otic placode markers PAX2 and PAX8 (Ealy et al., 2016). In parallel, a protocol utilising stromal cell-derived conditioned media was optimised by treating human PSCs with an initial FGF3/FGF10 exposure period, followed by the addition of EGF- and RA-supplemented conditioned media (Ding et al., 2016). This cocktail promoted the differentiation of hair cell-like cells that demonstrated morphological and electrophysiological profiles similar to those of cells differentiated by stromal cell co-culture (Ding et al., 2016). The characterisation of FGFs as factors necessary and sufficient for otic induction was evidenced by Chen et al. (2012), who established that initial FGF3/FGF10 treatment of human ESCs alone was able to produce two populations of otic progenitors: otic epithelial progenitors that give rise primarily to hair cells and supporting cells, and otic neural progenitors that give rise to neurons. Otic epithelial progenitors that were subsequently subjected to timed EGF and RA treatment differentiated into ATOH1^+^, POU4F3^+^ (BRN3C^+^) and MYO7A^+^ mechanosensitive hair cell-like cells (Chen et al., 2012). Subsequent work found that hair cell-like cell yields were improved when human iPSC-derived progenitors were treated with a Notch inhibitor instead of dual EGF/RA supplementation (Lahlou et al., 2018a). Alternative efforts have focused on expediting hair cell generation from human iPSCs by combining constitutive FGF3/FGF10 supplementation with transient TGF-β inhibition and Wnt modulation to induce POU4F3^+^ and MYO7A^+^ hair cell-like cells by culture day 13 (Lahlou et al., 2018b).

The development of these diverse approaches has resulted in remarkable progress in otic epithelial cultures; however, each protocol bears its own advantages and limitations. For example, although Oshima et al. (2010) observed hair cell functionality comparable to that of native cells, co-culture with foreign stromal cells may subvert normal developmental progression. The use of stromal cell-derived conditioned media (Ding et al., 2016; Ouji et al., 2012) can reduce this risk, but does not negate it, and conditioned media remain a source of variability that can impact reproducibility. The Ronaghi et al. (2014) protocol was able to generate hair cell-like cells from human ESCs without co-culture or conditioned media, but yields were reportedly low, and cells failed to develop organised stereocilia. Ealy et al. (2016) determined a requirement for RA to induce otic placode markers in their cultures, and thus it may be possible that RA would also improve hair cell differentiation. Indeed, in their protocol, Chen et al. (2012) derived hair cell-like cells with FGF, RA and EGF supplementation alone, although less than 1% of otic epithelial progenitors went on to develop stereocilia-like structures. Lahlou et al. (2018a) reported improved hair cell-like cell yields when EGF/RA supplementation was replaced with Notch inhibition (∼5% MYO7A^+^ cells versus ∼50% MYO7A^+^ cells); however, hair cell co-marker expression was not quantified, and stereocilia development and functionality were not assessed. Similarly, in their alternative protocol (Lahlou et al., 2018b), notable for its speed in hair cell-like cell induction and entirely monolayer-based, EB-free methods, mechanosensitivity remained undetermined. Interestingly, although supporting cell and neuronal cell marker genes were expressed in this culture, marker co-expression and organisation of these cells requires further investigation, and the observed expression of non-otic genes is indicative of heterogeneous or immature cell types.

### Manipulating gene expression to induce otic differentiation

Genetic approaches have also been used to drive otic differentiation. Inducible overexpression of *Atoh1* (*Math1*), a transcription factor highly expressed during early otic differentiation, in a mouse ESC line facilitated the differentiation of hair cell-like cells with stereocilia-like structures and some level of functionality (Ouji et al., 2013). In human iPSC-derived cultures, stereocilia maturity, according to morphology, density and MYO7A and ESPN expression, was improved when the overexpression of *ATOH1* was combined with that of transcription factors *RFX1* and *RFX3* (Chen et al., 2018a), which have been implicated in hair cell survival and are essential for hearing in mice (Elkon et al., 2015). In an EB-based protocol using mouse ESCs, combined overexpression of *Atoh1*, *Gfi1* and *Pou4f3* induced targeted hair cell differentiation without concomitant induction of neuronal or supporting cell types (Costa et al., 2015).

These protocols are useful in their ability to generate hair cell-like cells *in vitro* while minimising the variability associated with exogenous signalling manipulation*.* However, the direct activation of transcription factors in stem cells is likely to circumvent early or intermediate developmental phases, as has been previously observed (Costa et al., 2015). This would prevent the recapitulation of normal developmental progression and thus limit applications for developmental disease modelling or therapeutic trials in which developmental stages are relevant.

### Targeting supporting and neural cell induction in otic cultures

Besides sensory hair cell induction, other protocols have focused on the specification of epithelial supporting cells. These cells fulfil roles including regulation of planar cell polarity and synaptogenesis of hair cells during development (Gómez-Casati et al., 2010; Lee et al., 2012), and facilitation of ion flux and neurotransmitter recycling in the mature sensory epithelia (Glowatzki et al., 2006; Zhang et al., 2005; Zhu and Zhao, 2010). In non-human systems, supporting cells can facilitate hair cell regeneration by direct transdifferentiation (Box 1) and mitotic regeneration (Box 1) (Cox et al., 2014; Scheibinger et al., 2018).

In culture, cells expressing supporting cell markers have been generated from stem cell-derived ectodermal cells cultured on mouse-derived trypsin-resistant inner ear feeder cells. Ectodermal cells were initially induced in suspension culture from mouse iPSCs and ESCs by the simultaneous addition of Bmp4 and inhibition of Tgf-β (Fukunaga et al., 2016, 2021a), and from human iPSCs by the addition of insulin (Fukunaga et al., 2016, 2021a,b). In a protocol designed to generate specifically outer sulcus supporting cells, Hosoya et al. (2017) induced otic progenitor cells by adding an FGF signalling cocktail (FGF2/FGF3/FGF10/FGF19) and BMP4, alongside weak alkaline conditions. The resulting differentiated cells expressed high levels of the anion exchanger pendrin and other markers indicative of outer sulcus cells (Hosoya et al., 2017). Although the absence of an organised multi-cellular sensory epithelium in these cultures limits investigation of disease modelling on a whole-tissue scale, these protocols provide useful tools for studying pathogenesis at the cellular level.

In parallel to epithelial cell culture, an expanding body of work has focused on the generation of sensory otic neurons (Chen et al., 2012; Gunewardene et al., 2014; Hyakumura et al., 2019; Matsuoka et al., 2017; Nayagam et al., 2013). Neural and epithelial progenitors can be induced simultaneously (Chen et al., 2012), and mature PSC-derived neuron-like cells are able to synapse with rat explants of auditory hair cells (Hyakumura et al., 2019). However, a protocol for the 2D culture of an innervated sensory epithelium generated entirely from PSCs has yet to be defined.

### *In vitro*-derived cells as a source for cell therapies

A key advance made possible by 2D culture methods that generate otic progenitor cell types is the use of these cells for cell therapy, as explored in several proof-of-concept studies (Chen et al., 2018b; Li et al., 2003; Lopez-Juarez et al., 2019; Ouji et al., 2012, 2013; Takeda et al., 2018). In one study, otic progenitor cells that expressed pre-placodal transcriptional profiles were injected into embryonic chick ears. These cells were able to integrate into the developing epithelia, maturing into Myo7a^+^ and Espn^+^ hair cell-like cells in parallel with native chick epithelial cells (Li et al., 2003). In subsequent work, epithelial progenitors were incorporated into the organ of Corti in postnatal mice, where they differentiated into Myo7a^+^ hair cell-like cells and synapsed with native neurons (Chen et al., 2018b).

In another study, Dil-labelled human iPSC-derived otic progenitors were transplanted into the damaged adult guinea pig cochlea. This study showed that the labelled cells could migrate, integrate and differentiate *in vivo* (Lopez-Juarez et al., 2019). However, the possibility that the Dil label spread from the transplanted population to the remaining endogenous cells was not addressed. This is a common issue that has been observed in the eye field when labelled photoreceptor precursors are transplanted into the retina (Pearson et al., 2016). Not surprisingly, and reflecting a move towards 3D cultures, suspension cultures of mouse ESCs aggregated to form EBs and generated otic progenitors with greater efficiency than adherent cultures. These progenitors also possessed some capacity for incorporation into cochlear explants from postnatal ototoxin-injured mouse ears (Abboud et al., 2017). A proportion of these cells went on to express the hair cell marker Myo7a; however, synaptogenesis and functionality of these cells was not investigated.

Despite ongoing progress, the cell populations created in these 2D culture environments remain heterogeneous, and yields of the desired hair cell type remain low. Hair cell maturation has remained elusive, and simultaneous anterior and posterior placodal marker expression has been observed, in transitioning ectoderm cells, suggesting that 2D cultures may not faithfully recapitulate the *in vivo* developmental trajectory (Ealy et al., 2016). As we discuss next, the advent of 3D organoid cultures has helped to address some of these limitations.

## The advent of 3D differentiation culture systems

It has been suggested that the limited control that researchers have over otic differentiation in 2D cultures might be due to the absence of a defined microenvironment (Ealy et al., 2016). Although the addition of exogenous factors to a culture medium can approximate certain aspects of a tissue's microenvironment, the lack of other cell lineages might result in the absence of endogenous signalling activity that is as yet unknown. In 2D monolayers, the absence of a normal cellular architecture precludes the typical interactions that would normally occur between neighbouring cells and with the extracellular environment.

### Lessons learned from pioneering 3D culture studies

Landmark studies by Eiraku et al. (2008, 2011) were the first to introduce the *in vitro* differentiation of whole tissues in 3D that could more closely recapitulate *in vivo* development. In their 2008 study, EBs formed from mouse and human ESCs followed the cortical developmental trajectory, with tissue patterning being susceptible to FGF, Wnt and BMP signalling manipulation (Eiraku et al., 2008). In the 2011 study, these authors reported the *in vitro* generation of self-organised optic vesicles, derived from mouse ESCs, that went on to invaginate to form optic cups, a key structure in eye development (Eiraku et al., 2011). These studies paved the way for the use of suspension cultures to generate mature and complex 3D tissues. Interestingly, optic vesicle maturation into retinal vesicles was dependent on the manual excision of these structures from the neural EB, a process that limits the generation of large-scale samples for modelling and therapies, such as the isolation of sufficient cells for cell therapies. Indeed, in 2013, Gonzalez-Cordero et al. demonstrated the maturation of whole EBs that yield sufficient numbers of cells for proof-of-concept transplantation of mouse ESC-derived photoreceptor precursors (Gonzalez-Cordero et al., 2013).

The first 3D differentiation protocol for the inner ear was developed by Koehler et al. (2013) (Fig. 3B). Mouse ESCs were aggregated to form EBs in suspension and cultured in media containing Matrigel, triggering ectodermal differentiation. Following a similar stepwise induction process to those defined in the 2D protocols, aggregates were treated with a Tgf-β inhibitor and Bmp4 to induce NNE formation. Then, subsequent Bmp4 inhibition and Fgf2 activation stimulated the formation of PPE and of Pax8^+^/E-cadherin^+^ OEPD regions. This initial period of differentiation is crucial and requires the addition of factors at specific concentrations and time points to maintain the fine balance between the neuroectoderm and NNE, and subsequently the OEPD and other placodal regions. Differences between cell lines' endogenous expression levels of factors also necessitate the optimisation of protocols for specific ESC and PSC lines (DeJonge et al., 2016; Koehler et al., 2017). In particular, some cell lines have higher endogenous levels of BMP4 that can be sufficient to drive NNE differentiation without initial supplementation, whereas other cell lines require exogenous BMP4 to be added to the culture to prevent neuroectoderm differentiation (Koehler et al., 2017).

In these mouse stem cell-derived 3D cultures, otic vesicles form autonomously within the aggregate, following OEPD formation. Prolonged culture eventually generates organised sensory epithelia that contain numerous Myo7a^+^ and Pou4f3^+^ hair cells. These cells express markers for ribbon synapses (Box 1), such as Ctbp2, and are innervated by Tuj1^+^ (Tubb3^+^) and Nefl^+^ neurons, indicating the attainment of developmental milestones not previously reported in 2D cultures (Koehler et al., 2013). In addition to otic tissues, mouse ESC-derived organoids contain cells that express markers typical of other lineages, including the mesendoderm and neuroectoderm, and later mesenchymal tissues and cartilage.

Subsequent research determined that treating whole aggregates with the Wnt agonist CHIR99021 immediately prior to the onset of Pax2 expression can increase the number of Pax2^+^/Pax8^+^ prosensory vesicles and improve hair cell yields (DeJonge et al., 2016). Hair cells produced according to this protocol exhibited mature electrophysiological profiles, demonstrating mechanosensitivity levels comparable to those in postnatal mice (Liu et al., 2016). Importantly, in these cultures, maturation of hair cells containing sensory epithelia was not dependent on mechanical manual dissection.

In 2017, the protocol of DeJonge et al. (2016) with Wnt agonist supplementation was adapted to human PSCs to generate inner ear organoids with innervated sensory epithelia by day 60 in culture (Koehler et al., 2017). Neurites extending from NEFL^+^ and NEFH^+^ neurons were observed contacting hair cells in these organoids, and puncta positive for the pre-synaptic markers CTBP2 and synaptophysin indicated the formation of ribbon synapses. Mature organoids exhibited electrophysiological profiles that were reminiscent of native human vestibular hair cells.

### Alternative 3D culture strategies

In recent years, several alternative 3D culture strategies have been devised. Mattei et al. (2019) reported the production of human ESC- and iPSC-derived organoids that contained mature hair cells using a rotary cell culture system in which aggregates were suspended in rotating media, enabling constant motion while minimising shearing forces. In the resulting organoids, helium ion microscopy and microcomputed tomography indicated the presence of crystalline structures overlying the surface of hair cells, suggestive of otoconia formation. Hocevar et al. (2021) adapted the protocols of Koehler et al. (2013) and DeJonge et al. (2016) to generate mouse ESC-derived otic vesicles that were mechanically sifted out from whole aggregates. Isolated vesicles expanded to form cyst-like structures and autonomously generated hair cell-containing sensory epithelia when embedded in Matrigel.

In addition to PSC-derived organoids, 3D cultures have also been generated from somatic inner ear cells. LGR5^+^ supporting cells from neonatal and adult mice and an adult human, and cochlear greater epithelial ridge (Box 1) cells from neonatal mice have been used to produce organoids that generate hair cell-like and supporting cell-like cells without the induction of other cell types (Kubota et al., 2021; McLean et al., 2017). Similar results have been achieved using mouse embryonic fibroblasts, in which direct overexpression of otic lineage genes, including the transcription factors *Sox2* and *Six1*, and the transcriptional co-activator *Eya1*, generated sensory epithelial progenitors and subsequent hair cell-like cells (Yang et al., 2021).

### Summary and synergy

Stem cell-derived inner ear organoids break new ground for otic cultures. They contain an organised cytoarchitecture more akin to the *in vivo* developing ear than can be achieved in 2D monolayers, allowing for more accurate developmental recapitulation. However, the variable reproducibility of the system (Carpena et al., 2021; Schaefer et al., 2018), and the laborious need to optimise protocols specific to each pluripotent stem cell line (DeJonge et al., 2016; Koehler et al., 2017), has resulted in a small number of studies using 3D inner ear organoids to date. Furthermore, the absence of definitively characterised cochlear structures and cell types, as only vestibular organoids have been generated to date (Koehler et al., 2013, 2017), and a lack of clarity around the identity and organisation of the cell types present in developing and mature aggregates, has limited translational applications of current 3D inner ear organoid protocols.

## Modelling inner ear diseases

Genetic auditory and vestibular diseases can be modelled in 2D and 3D cultures by employing gene-editing tools to induce pathogenic mutations in PSCs or produce isogenic controls from patient-derived PSCs. In parallel, acquired dysfunction can be investigated by exposing cultured cells and organoids to infectious or damaging agents, including pathogens and ototoxic drugs.

### Inherited deafness – what have we learned from *in vitro* models?

**Fig. 4.**
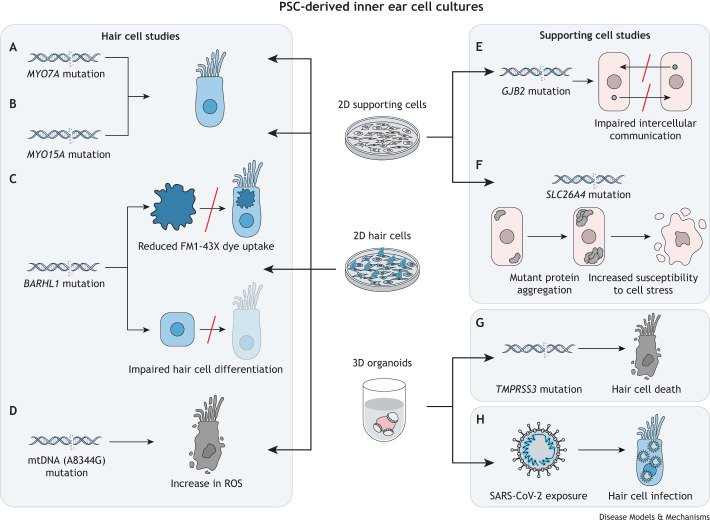
**Schematic overview of disease modelling studies using PSC-derived inner ear cultures.** This schematic depicts different disease-modelling studies that used 2D or 3D otic culture protocols to explore the pathophysiology of a genetic mutation or infection that affects auditory and/or vestibular function. (A-D) Phenotypes observed in hair cell-like cells in 2D cultures derived from PSC lines bearing pathogenic mutations. (E,F) Phenotypes observed in supporting cell-like cells in 2D cultures derived from PSC lines bearing pathogenic mutations. (G) The phenotype observed in inner ear organoid cells derived from a PSC line with a pathogenic mutation in *TMPRSS3*. (H) The phenotype observed in inner ear organoid cells and hair cell-like cells in 2D culture following exposure to the infectious SARS-CoV-2 virus. mtDNA, mitochondrial DNA; PSC, pluripotent stem cell; ROS, reactive oxygen species.

Several studies have used PSC-derived hair cell-like cells to investigate the pathophysiology caused by variants in deafness-associated genes. *MYO7A*, which has purported roles in stereocilia development (Boëda et al., 2002; Prosser et al., 2008) and mechanotransduction (Li et al., 2020), has been implicated in two forms of non-syndromic deafness (DFNA11 and DFNB2; Box 1) (Liu et al., 1997; Weil et al., 1997) and in Usher syndrome (Box 1) type 1B (Weil et al., 1995). Tang et al. (2016) derived iPSCs from the urinary cells of a patient with autosomal-recessive hereditary deafness caused by compound heterozygous mutations in *MYO7A*. Cells were successfully differentiated in 2D culture to produce PAX2^+^, PAX8^+^ and SOX2^+^ otic epithelial progenitor cells and subsequent ATOH1^+^, POU4F3^+^ and MYO7A^+^ hair cell-like cells. Electron microscopy revealed that stereocilia were morphologically abnormal across all cell lines, likely due to limitations of the 2D culture system. However, in comparison to control cells, the stereocilia of *MYO7A*-deficient cells were further deformed and non-cohesive. These hair cell-like cells showed reduced functionality, as demonstrated by the uptake of FM1-43X dye and electrophysiological profiling, compared to control cells. These findings indicate that MYO7A is required for the development of mechanosensitive hair cells, possibly by regulating stereocilia formation, as has been described in mouse models (Boëda et al., 2002; Prosser et al., 2008) (Fig. 4A). In another study, iPSCs were generated from dermal fibroblasts of a patient with recessive inherited profound hearing loss, who had compound heterozygous mutations in myosin XVA (*MYO15A*) (Chen et al., 2016), which is associated with the transport of stereocilia tip-related proteins and regulation of stereocilia height (Belyantseva et al., 2005; Manor et al., 2011). The differentiation of *MYO15A*^−/−^ hair cell-like cells from this patient was achieved in 2D; however, some cells formed syncytia or died. In those cells that survived, stereocilia were significantly shorter, and cells demonstrated impaired functionality, as demonstrated by reduced FM1-43X dye uptake and diminished electrophysiological conductance. The results of these studies suggest that pathogenic mutations in *MYO7A* and *MYO15A* permit normal otic differentiation, but impair stereocilia development, which precludes typical mechanotransduction (Fig. 4B). In Myo7a- and Myo15a-deficient mouse models, stereocilia defects were similarly observed and suggested to be the result of misregulation of stereocilia height and organisation due to a lack of Myo7a- and Myo15a-dependent protein transport (Belyantseva et al., 2005; Boëda et al., 2002; Manor et al., 2011; Prosser et al., 2008). Corroboration with phenotypes observed in mouse models serves to validate PSC-derived cultures as a disease modelling system and provides evidence that the functions of MYO7A and MYO15A are conserved in humans.

To study the roles of the deafness-associated BARHL1 homeodomain transcription factor, CRISPR/Cas9 was used to induce a targeted mutation in *Barhl1* in mouse ESCs (Zhong et al., 2018). The resulting *Barhl1*-mutated cells differentiated into otic epithelial progenitor cells in 2D culture, as demonstrated by quantitative reverse transcription polymerase chain reaction (RT-PCR) and immunocytochemistry for otic markers. However, their subsequent differentiation into hair cell-like cells was impeded, as shown by the reduced expression of hair cell markers Pou4f3, Myo7a and Espn, and decreased FM1-43X uptake. According to quantitative RT-PCR data, *Barhl1* expression commenced at approximately day 9 in culture, and RNA sequencing at this time point was used to identify putative Barhl1 target genes. Notably, two potential targets included genes previously associated with sensorineural hearing loss: *Clic5*, which is a member of a chloride ion channel family implicated in autosomal recessive progressive hearing loss with vestibular dysfunction (Seco et al., 2015), and *Ush1g*, which encodes a scaffolding protein associated with Usher syndrome (Weil et al., 2003). This suggests that Barhl1 might direct hair cell differentiation by regulating expression of these otic effectors. To further elucidate these mechanisms, Hou et al. (2019) mutated the E3 site of the E-box in the *Barhl1* 3′ enhancer, which contains a putative Atoh1 binding site. Using the same mouse ESC-derived *in vitro* 2D culture system as Zhong et al. (2018), they observed a similar phenotype to the loss-of-function mutation, indicating that Atoh1 is required for *Barhl1* activation. These findings support previous reports that implicate Atoh1 as a regulator of *Barhl1* expression (Chonko et al., 2013), and clarify the interactions that drive otic development and mediate BARHL1-related pathogenesis (Fig. 4C). These studies highlight the amenability of PSCs to gene editing to investigate both gene function and regulation.

In a model of sensorineural hearing loss in myoclonus epilepsy with ragged-red fibres (MERRF) syndrome (Box 1), iPSC lines were derived from siblings with an A8344G substitution mutation in their mitochondrial DNA (mtDNA) (Chen et al., 2018a). These iPSC lines were differentiated in 2D culture into hair cell-like cells, and their differentiation progressed normally compared to isogenic controls. However, reactive oxygen species (ROS) accumulated in both the diseased iPSCs and differentiated hair cell-like cells, as measured by the uptake of CellROX dye. Antioxidant gene expression was also increased in both cell types, indicating activated ROS clearance pathways. Increased ROS production in A8344G iPSC-derived cells has previously been observed in cardiomyocyte and neural progenitor cells (Chou et al., 2016), suggesting that increased susceptibility to ROS production may be ubiquitous across multiple tissues in MERRF patients. Scanning electron microscopy revealed that A8344G hair cell-like cells had shorter stereocilia and more singular cilium structures compared to the isogenic control. When differentiation was paired with overexpression of *ATOH1*, *RFX1* and *RFX3* transcription factor genes, which improved stereocilia maturation across cell lines, A8344G cells generated stereocilia bundles at a reduced density in comparison to the control. Impaired stereocilia formation may therefore play a role in MERRF syndrome-associated hearing loss (Fig. 4D). However, further investigation is required to determine whether this phenotype is related to oxidative stress.

In cases in which hearing loss is mediated by supporting cell defects, researchers have employed 2D PSC-derived cultures of non-sensory cells. To investigate deafness caused by mutations in *GJB2*, encoding the gap junction protein connexin 26, Fukunaga et al. (2016) devised a protocol to differentiate mouse iPSCs into supporting cell-like cells, as discussed above. In healthy cells, connexin 26 associates with connexin 30 (encoded by *GJB6*) to form gap junctions between supporting cells (Ahmad et al., 2003; Jagger and Forge, 2006). Differentiated connexin 26-deficient cells derived from mouse iPSCs exhibited fragmented connexin 30 aggregates in comparison to organised linear connexin 26–connexin 30 structures that lined cell–cell borders in control cells. This phenotype was replicated when iPSCs derived from peripheral blood mononuclear cells of two siblings with profound hearing loss and homozygous *GJB2* mutations were differentiated into supporting cell-like cells (Fukunaga et al., 2021b). Reduced intercellular communication was confirmed using a Lucifer Yellow scrape loading assay, suggesting that pathogenicity stems from impaired gap junction formation preventing communication between supporting cells (Fig. 4E) (Fukunaga et al., 2021b). Here, the conserved disease phenotypes in cultures produced from a mouse ESC line (Fukunaga et al., 2021a) and multiple mouse iPSC (Fukunaga et al., 2016) and patient iPSC (Fukunaga et al., 2021b) lines indicate a level of reliability in these PSC-derived disease modelling systems. However, further investigation is required to definitively characterise whether *GJB2*-related deafness is caused by impaired intercellular communication, and how affected supporting cells might interact with other cell types of the sensory epithelium.

To investigate hearing loss in Pendred syndrome (Box 1), a disease caused by mutations in *SLC26A4*, encoding the pendrin anion exchanger (Everett et al., 1997), Hosoya et al. (2017) developed a novel protocol to generate outer sulcus cell-like cells from patient-derived iPSCs. No difference in anion exchange activity was detected in diseased cells, suggesting that pathology was not caused by loss of pendrin anion exchange capacity. Relatively high numbers of cells containing pendrin aggregates were observed, and these aggregates co-localised with ubiquitin and LC3B (MAP1LC3B), indicating the activation of the ubiquitin–proteasome degradation and/or autophagy pathways. When proteasome degradation was inhibited with epoxomicin treatment, the rates of caspase-3 expression and cell death increased relative to the control cell line, indicating a greater susceptibility to cell stress. This might result in supporting cell death, subsequent hair cell degeneration and progressive hearing loss *in vivo* (Fig. 4F) (Hosoya et al., 2017), which could be confirmed in future studies using models containing both cochlear supporting and hair cells. This study highlights the utility of PSCs for disease modelling where alternative models are lacking, as has historically been the case for Pendred syndrome (Lu et al., 2014).

To study hearing loss associated with TMPRSS3, a transmembrane serine protease implicated in recessive non-syndromic hearing loss, within intact sensory epithelia, Tang et al. (2019) employed mouse ESC-derived organoids as a disease model. In organoids derived from both mutant and knockout *Tmprss3* lines, the temporal expression of otic markers indicated their normal developmental progression, and FM1-43FX dye uptake revealed that differentiated knockout hair cells retained functionality. However, following organoid maturation, upregulated caspase-3 expression was detected in the sensory epithelia at a developmental time point that is roughly equivalent to 2-week old mice, a phenotype similar to that observed in *Tmprss3* mutant mice. Knockout organoids analysed prior to the onset of detectable caspase-3 exhibited a reduced number of calcium-activated K^+^ channels (BK channels), similar to that previously reported in *Tmprss3* mutant mice (Molina et al., 2013). Single-cell RNA sequencing of knockout organoids identified multiple differentially expressed genes with products that are reported to interact with the BK channel subunit, KCNMA1. RNA-sequencing data also indicated reduced expression of calcium-binding genes and increased expression of extracellular matrix (ECM)-associated genes, an association further supported by the localisation of Tmprss3 to the cell membrane. In view of their findings, the authors inferred that Tmprss3 is required to maintain hair cell survival after differentiation and that it is involved in maintaining calcium homeostasis by interacting with BK channels and regulating or organising the ECM. Importantly, these *in vitro* observations reflect reported *in vivo* mouse phenotypes, validating the inner ear organoid as a suitable model in which to recapitulate, and thus further investigate, *Tmprss3-*related pathogenesis (Fig. 4G)*.*

### PSC-derived inner ear culture as a model for acquired audiovestibular dysfunction

Although most PSC-derived, culture-based disease modelling studies have so far focused on inherited diseases, a recent study used both 2D and 3D cultures to investigate SARS-CoV-2 inner ear infection, following a clinical evaluation of audiovestibular dysfunction in ten patients with COVID-19 (Jeong et al., 2021). Human iPSCs were used to generate monolayers of either otic prosensory cells (OPCs), expressing otic placodal markers, or Schwann cell precursors (SCPs). Both cell populations expressed ACE2, a receptor protein for SARS-CoV-2, and the viral entry co-factors TMPRSS2 and FURIN. To investigate whether cells could be directly infected with SARS-CoV-2, cultures were incubated with virus-containing media. Although infection of SCPs was rare, approximately 26% of OPCs from one cell line contained detectable nucleoproteins 48 h after inoculation, and viral RNA was detectable for up to 72 h after infection, indicating vulnerability in these cells to sustained infection when directly inoculated. In cultured organoids, ACE2 could be detected in hair cells and Schwann cells but not in neurons, in line with expression patterns observed in human vestibular explants. Immunostaining for double-stranded RNA (dsRNA) in organoids inoculated with virus-containing media indicated that infection occurred predominantly in hair cells. The expression of SARS-CoV-2 entry factors and the vulnerability of hair cells to artificial SARS-CoV-2 inoculation offer preliminary insights into how COVID-19-associated audiovestibular dysfunction might be mediated by hair cell infection (Fig. 4H). The testing of other acquired conditions in inner ear organoids will be crucial for further developing therapies for hearing loss and vestibular dysfunction.

## Trialling therapeutics

**Fig. 5.**
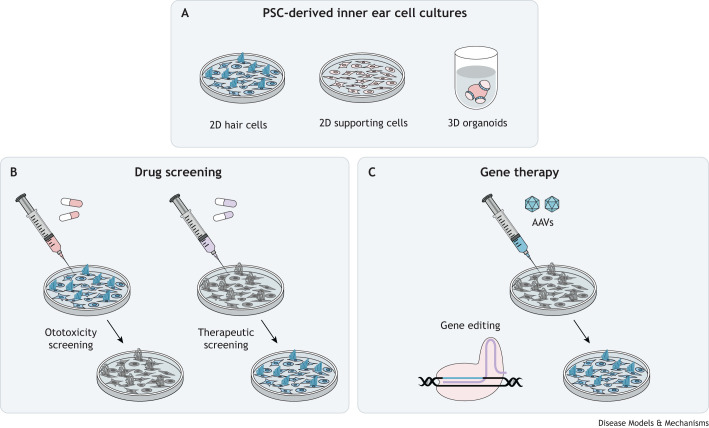
**Applications for inner ear cultures in drug screening and gene therapy development.** (A) PSC-derived 2D (hair and supporting cells) and 3D inner ear cultures (organoids) can be used to screen drugs and develop gene therapies. (B) Cultures of healthy inner ear cells can be used to screen drugs and compounds, such as aminoglycoside antibiotics, for off-target ototoxicity. Cultures of diseased or damaged inner ear cells can be used to investigate the therapeutic efficacy of drugs. (C) Cultures derived from PSCs that carry disease-associated pathogenic mutations can be used for gene therapy using viral vectors, such as AAVs, to investigate vector tropism in gene therapy development, and to trial novel gene therapy approaches, such as gene editing. AAV, adeno-associated viral vector; PSC, pluripotent stem cell.

Inner ear disorders are exceedingly common, yet hearing aids and cochlear implants are often the only means of treatment, and, although they have offered significant benefit, they are unable to fully restore hearing. There is, therefore, a pressing need to develop new therapeutic strategies for the inner ear, and a parallel requirement for model systems to investigate efficacy and toxicity in human cells. Although this Review focuses on the applications of PSC-derived inner ear cultures in disease modelling and as platforms to trial therapeutic agents (Fig. 5), it is worth noting that a separate body of work has made marked progress in elucidating the mechanisms of endogenous sensory regeneration in animal models and in devising strategies to induce regeneration in humans for functional restoration (Box 2). As this field progresses, PSC-derived cultures may also provide a useful tool to investigate regenerative approaches *in vitro.*

**Box 2. Therapeutic regeneration in the otic sensory epithelia**

Fish, birds and, to an extent, neonatal mice are able to regenerate lost hair cells from supporting cell populations (Cox et al., 2014; Ma et al., 2008; Scheibinger et al., 2018), leading researchers to investigate whether the signalling systems and transcription factors involved can be manipulated to artificially induce endogenous cell types to regenerate for therapeutic benefit. For example, chemically or genetically activating Wnt and inhibiting Notch signalling can increase supporting cell proliferation and hair cell differentiation in both damaged (Bramhall et al., 2014; Mizutari et al., 2013; Wu et al., 2016) and undamaged mouse epithelia (Li et al., 2015; Wu et al., 2016). Activating otic transcription factors, such as Atoh1, is an alternative approach that has been used in supporting cells to drive direct transdifferentiation; however, Atoh1-driven regenerative capacity diminishes with age and fails to yield fully mature hair cells (Kelly et al., 2012; Liu et al., 2012, 2014). Subsequent work has focused on identifying combinations of transcription factors to improve differentiation; for example, overexpression of Gfi1 and Pou4f3 together with Atoh1 was sufficient to drive hair cell differentiation directly from mouse embryonic stem cells (Costa et al., 2015). More recently, overexpression of Atoh1/Gfi1/Pou4f3 and Six1 in mature murine cochlear supporting cells was able to induce functional hair cell-like cells *in vitro* (Menendez et al., 2020). In *in vivo* adult mice cochlea, combined Atoh1/Gfi1 overexpression similarly enabled differentiation of supporting cells into hair cell-like cells (Lee et al., 2020b). In both studies, transdifferentiation occurred more efficiently when induced with combined transcription factor activation in comparison to Atoh1 overexpression alone. However, the derived hair cell-like cells still lacked maturity in relation to transcriptional and electrophysiological profiles (Menendez et al., 2020), and stereocilia organisation and synaptogenesis (Lee et al., 2020b), indicating that further studies are needed before this approach can be translated to the clinic.

Currently, pioneering 2D PSC-derived disease models provide a basis for drug design and testing. For example, after observing autophagy activation in supporting cell cultures derived from Pendred syndrome patients, Hosoya et al. (2017) investigated whether artificially driving autophagy with rapamycin and metformin could ameliorate cell death induced by the proteasome degradation inhibitor, epoxomicin. This treatment successfully increased cell viability and reduced caspase-3 expression, validating these drugs as potential treatment options. Rapamycin also demonstrated efficacy in increasing cell viability in long-term culture for up to 42 days, highlighting the potential of this drug to preserve cochlear supporting cells (Hosoya et al., 2019). Future work, including investigations in organoids, could help to establish the global effect of such treatments in the whole inner ear environment. Protocol optimisation is also likely to increase cell yields, enabling larger-scale, drug-screening research, for therapeutic benefit and to investigate the off-target ototoxicity of medications, such as aminoglycoside antibiotics (Alharazneh et al., 2011).

Similarly, PSC-cultures offer a useful platform for gene therapy trials to facilitate personalised approaches. Thus far, studies using inner ear cultures have treated the iPSCs prior to differentiation, offering proof of concept that the devised therapies are able to prevent the manifestation of disease phenotypes before they arise during development. In their model for Pendred syndrome, Hosoya et al. (2017) transfected iPSC cells with transcription activator-like effector nucleases (TALENs) to repair a causal pathogenic substitution mutation in one of their iPSC lines. The repaired cells exhibited reduced levels of intracellular pendrin protein aggregates at a level similar to that in control lines, and lower rates of epoximicin-induced cell death. Similarly, CRISPR/Cas9 has been used to correct substitution mutations in *MYO7A* and *MYO15A* in iPSCs generated from deaf patients, using a homology-directed repair approach. In the gene-edited cells, stereocilia morphology and hair cell-like cell electrophysiological capacity were restored, relative to control lines (Chen et al., 2016; Tang et al., 2016). The above studies highlight the applicability of stem cell-derived cultures as a means to develop personalised medicine approaches that are specific to a patient's genome. However, further work is needed to investigate efficacy in targeted cell types, and not solely in their stem cell precursors, to enable progression towards clinical usage.

Progress in stem cell-derived organoid cultures promises to facilitate the pre-clinical testing of gene therapy *in vitro* in human cells and tissues, but the clinical translation of these findings will require concurrent research to overcome challenges in application. Although accessibility to the cochlear and vestibular tissues is limited, it is possible to directly administer gene therapies to affected cells *in situ*. Many studies have now demonstrated proof of concept for gene therapies in mouse models (Akil et al., 2019; Gu et al., 2021; György et al., 2019b; Nist-Lund et al., 2019; Taiber et al., 2021; Yeh et al., 2020). In recent years, adeno-associated viral vectors (AAVs) have emerged as the predominant vector of choice for human gene therapies, and AAV capsids have been developed to target specific inner ear cell types, including the AAV2/Anc80L65, AAV2/7m8 and AAV9/PHP.B vectors (György et al., 2019a; Isgrig et al., 2019; Landegger et al., 2017). Importantly, these have demonstrated efficacy in animal models of injury and disease (Akil et al., 2019; Gu et al., 2021; György et al., 2019b; Nist-Lund et al., 2019; Taiber et al., 2021; Yeh et al., 2020), but challenges in attaining high efficiency of transduction and gene editing in all target cell types and in all regions of the cochlea persist (Gu et al., 2021; György et al., 2019b; Nist-Lund et al., 2019; Yeh et al., 2020).

An alternative therapeutic approach to gene therapy is transplantation of stem cell-derived otic sensory epithelial cells or progenitor cells, as has been demonstrated in animal models (Chen et al., 2018b; Li et al., 2003; Lopez-Juarez et al., 2019; Ouji et al., 2012, 2013; Takeda et al., 2018). Successful engraftment has been observed in mouse cochleae after round window (Box 1) injection (Chen et al., 2018b), and guinea pig cochleae after cochleostomy (Box 1) (Lopez-Juarez et al., 2019), highlighting these as feasible delivery routes in mammalian systems. However, further investigation is required to determine whether transplanted cells can survive long term and contribute functionality, to understand transplant immunogenicity, and to optimise the numbers of cells engrafting and integrating into the native cellular architecture of the targeted sensory epithelia. These are all issues that have previously been encountered in animal models (Chen et al., 2018b; Lopez-Juarez et al., 2019; Takeda et al., 2018).

A key issue in translating these gene and cell therapies to the clinic is the time window in which treatment remains viable, particularly in cases where progressive disease may result in degeneration of the sensory epithelia before birth. In advanced cases of disease, cell transplantation is one of the only viable treatment options. Thus far, many gene therapy trials for inner ear disease have used neonatal mice (György et al., 2019b; Taiber et al., 2021; Yeh et al., 2020), and problems have been encountered in achieving effective transduction at more mature time points (Nist-Lund et al., 2019). However, successful restoration of hearing has been reported in mature mice in a model of DFNB9 (Box 1) following replacement of the *Otof* gene, encoding a calcium sensor involved in neurotransmitter exocytosis (Akil et al., 2019). Nevertheless, more disease-specific research will be needed to verify therapeutic efficacy at different stages of maturity in order to facilitate clinical translation of gene and cell therapies.

## Challenges and limitations – what's next?

PSC-derived inner ear culture research will need to surmount several challenges before clinical applications can be expanded. Variable outcomes have been reported from cultures generated from the same protocols, highlighting a need for improved reproducibility. For example, Schaefer et al. (2018) found that modified concentrations of knockout serum replacement and FGF2 were required to induce organoids relative to the concentrations reported by Koehler et al. (2013). The incidental induction of skin organoids that contain keratinocyte-like cells has also been reported when using otic organoid protocols. This induction occurs at varying efficiencies, depending on the stem cell line used and the endogenous expression levels of factors, such as BMP4 (Koehler et al., 2013; Lee et al., 2018, 2022). Indeed, recent skin organoid protocols have been based on original otic organoid protocols (Lee et al., 2018, 2020a, 2022). Another study, using a protocol derived from Koehler et al. (2013) and DeJonge et al. (2016), observed the unintended induction of neural-, cardiac- and follicular skin-like organoids, in addition to inner ear organoids (Carpena et al., 2021).

**Fig. 6.**
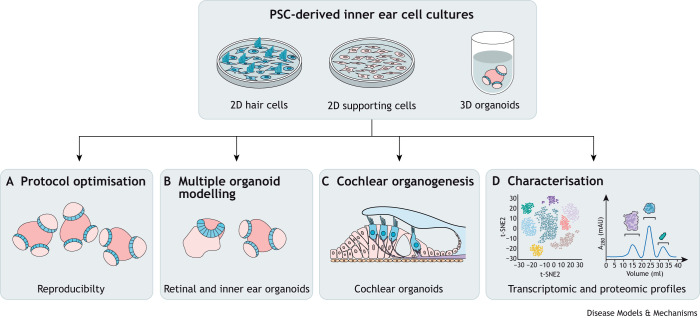
**Possible future research directions for PSC-derived inner ear cultures.** (A) Optimisation should improve the reproducibility of specific culture protocols, improving their utility for future research. (B) Co-culturing of different organoid systems, such as the retina and inner ear, will facilitate disease modelling in multiple tissue types simultaneously. (C) The development of protocols able to generate organoids with cochlear cell types would support cochlear-specific research and therapeutic development. (D) More detailed characterisation, including transcriptomic and proteomic profiling, should also improve our understanding of how closely *in vitro* cultures recapitulate *in vivo* development. A_280_, absorbance at 280 nm; mAU, milli absorbance unit; PSC, pluripotent stem cell; t-SNE2, t-distributed stochastic neighbour embedding 2.

Variability between cell lines and the difficulty in achieving the balance necessary to generate the otic placode regions in organoids have perhaps contributed to the lack of studies that model inherited inner ear diseases using human iPSC-derived 3D organoids. Currently, heterogeneity between PSC lines can be partially mitigated within individual studies by implementing isogenic control lines generated with gene-editing technologies, and by cell line-dependent protocol optimisation (Fig. 6A). However, the protocols themselves need to be refined and standardised to improve reproducibility and to maximise their utility for complex applications, such as those involving multiple organoid systems (Fig. 6B). For example, in the future, it might be possible to pair retinal and inner ear organoids to elucidate pathophysiology of deaf-blindness syndromes, such as Usher syndrome, and to enable the development of new treatments effective in both organs.

The inability of organoid culture protocols to generate cochlear cell types is another limitation to their versatility for tissue-specific modelling (Fig. 6C). Current evidence indicates that the sensory cells generated in organoids are limited to type I and II vestibular hair cells (DeJonge et al., 2016; Koehler et al., 2013, 2017; Liu et al., 2016; Schaefer et al., 2018). Putative cochlear hair cells have been reported once from organoid culture (Jeong et al., 2018), but these observations have not been validated by thorough cell type characterisation. The vestibular identity of organoids does not necessarily preclude their utility in cochlear disease modelling, as vestibular cell types can be used as a proxy for cochlear cell types, which is an approach taken in previous studies (Jeong et al., 2021; Tang et al., 2019). Further, they provide a viable platform for modelling vestibular dysfunction, an often overlooked comorbidity that frequently occurs with inherited hearing loss, for example in association with mutations in *CLIC5* (Seco et al., 2015), *COCH* (Box 1) (Robertson et al., 1998) and *ESPN* (Box 1) (Naz et al., 2004). Although 2D PSC-derived cultures and 3D somatic cell-derived cultures offer alternative options for modelling disease in cochlear cell types, a comprehensive understanding of pathology in inherited and acquired hearing impairments and how intact tissues may respond to putative therapeutics would benefit from the development of cochlear cell-bearing organoids. It has been suggested that cochlear specification *in vitro* may be induced by the addition of ventralising signalling factors, such as SHH (Schaefer et al., 2018), considering its activity in cochlear formation *in vivo* (Riccomagno et al., 2002). However, further work is required to investigate this possibility.

Further research using inner ear organoids will also benefit from a more thorough understanding of how closely they mimic the *in vivo* organ (reviewed in van der Valk et al., 2021). Notably, a study using a Venus-reporter construct to investigate *Fbxo2* expression in mouse ESC-derived organoids reported discrepancies in expression patterns in both hair cells and supporting cells in comparison to those in transgenic mice bearing the same reporter construct, indicating the potentially inaccurate recapitulation of cell specification and differentiation (Hartman et al., 2018). Investigating this possibility will require organoids to be characterised throughout their development. A comprehensive comparison of transcriptomic and proteomic profiles will help to verify the ability of organoids to mimic *in vivo* organogenesis (Fig. 6D). This research will be useful for understanding the capacity and limitations of organoids as model systems. Moreover, the elucidation of the molecular mechanisms of health and disease using these techniques might also provide us with new disease biomarkers.

## Conclusion

Despite ongoing challenges, PSC-derived inner ear cultures have already demonstrated potential for disease modelling and therapeutic trials, and continued research and protocol optimisation is likely to improve their applications and outcomes. Stem cell differentiation cultures, particularly in the form of organoids, bridge the gap from animal models into human systems prior to clinical trials. Prolonged culture periods will also provide new avenues of study into developmental and progressive diseases, where tissues can be analysed over developmental time and into maturity. The use of patient-derived cultures can facilitate the evaluation of gene therapy efficacy, a possibility that has been trialled in other model systems, such as the eye (Kruczek et al., 2021). Inner ear organoids could also be used to assess vector tropism in target cell types and predict off-target transduction in other inner ear cell types.

Despite the progression described here, the field of disease modelling using PSC-derived inner ear cultures, particularly organoid cultures, remains in its infancy and exciting times are still to come.
